# Negative Regulation of the Mis17-Mis6 Centromere Complex by mRNA Decay Pathway and EKC/KEOPS Complex in *Schizosaccharomyces pombe*

**DOI:** 10.1534/g3.119.400227

**Published:** 2019-04-09

**Authors:** Xingya Xu, Norihiko Nakazawa, Li Wang, Orie Arakawa, Mitsuhiro Yanagida

**Affiliations:** G0 Cell Unit, Okinawa Institute of Science and Technology Graduate University, Onna-son, Okinawa 904-0495, Japan

**Keywords:** Mis17-Mis6, suppressor screen, EKC/KEOPS, Exo2, Cnp1/spCENP-A

## Abstract

The mitotic kinetochore forms at the centromere for proper chromosome segregation. Deposition of the centromere-specific histone H3 variant, spCENP-A/Cnp1, is vital for the formation of centromere-specific chromatin and the Mis17-Mis6 complex of the fission yeast *Schizosaccharomyces pombe* is required for this deposition. Here we identified extragenic suppressors for a Mis17-Mis6 complex temperature-sensitive (ts) mutant, *mis17-S353P*, using whole-genome sequencing. The large and small daughter nuclei phenotype observed in *mis17-S353P* was greatly rescued by these suppressors. Suppressor mutations in two ribonuclease genes involved in the mRNA decay pathway, *exo2* and *pan2*, may affect Mis17 protein level, as *mis17* mutant protein level was recovered in *mis17-S353P exo2* double mutant cells. Suppressor mutations in EKC/KEOPS complex genes may not regulate Mis17 protein level, but restored centromeric localization of spCENP-A/Cnp1, Mis6 and Mis15 in *mis17-S353P*. Therefore, the EKC/KEOPS complex may inhibit Mis17-Mis6 complex formation or centromeric localization. Mutational analysis in protein structure indicated that suppressor mutations in the EKC/KEOPS complex may interfere with its kinase activity or complex formation. Our results suggest that the mRNA decay pathway and the EKC/KEOPS complex negatively regulate Mis17-Mis6 complex-mediated centromere formation by distinct and unexpected mechanisms.

A centromere is a part of a chromosome and it plays a crucial role in equal sister chromatid separation. Kinetochore is a protein complex assembled at each centromere, it associates with microtubules and motor proteins that segregate the divided sister chromatids toward the spindle poles in mitosis (Mitchison and Salmon 1992). Kinetochores in metaphase chromosomes must be bioriented toward opposite spindle poles to ensure correct sister chromatid separation (Rieder *et al.* 1995).

Centromeres of the fission yeast *S. pombe*, contain a series of outer repeats (otr) that flank inverted inner repeats (imr) surrounding a central non-repetitive region (cnt) (Takahashi *et al.* 1992). CENP-A is the kinetochore-specific histone H3 of kinetochore-specific nucleosomes. The fission yeast CENP-A-like kinetochore-specific histone H3, spCENP-A (designated Cnp1), is present in the central domain (imr and cnt) of the centromeres, but not in the outer repetitive heterochromatic regions (Chen *et al.* 2003; Takahashi *et al.* 2000).

Previous analysis of *mis6*, *mis12*, and *cnp1* has established that large and small nuclei phenotype is the hallmark of mutations in authentic kinetochore components (Goshima *et al.* 1999; Saitoh *et al.* 1997; Takahashi *et al.* 2000). More than 1,000 ts mutants have been observed at the restrictive temperature (36°) using fluorescence microscopy after DAPI staining (4,6-diamidino-2-phenylindole, a fluorescent probe for DNA) and five more fission yeast centromere proteins (designated Mis14–18), ts mutants that exhibited the large and small nuclei phenotype at the restrictive temperature, have been identified (Hayashi *et al.* 2004). Mis19 and Mis20, which directly interact with Mis16 and Mis18, were identified too (Hayashi *et al.* 2014; Hirai *et al.* 2014). Mis16 and Mis18 form a complex and maintain the deacetylated state of histones specifically in the central core of centromeres. Mis16 and Mis18 are the most upstream factors in kinetochore assembly, as they can associate with centromeric DNA in all kinetochore mutants, except *mis18* and *mis16* mutants, respectively. Mis6 and Mis15–20 are all part of the CENP-A recruitment pathway, while Mis12 and Mis14 are not. Mis6, Mis15, and Mis17 form an evolutionarily conserved complex that also includes Sim4, and Mal2 (Jin *et al.* 2002; Kerres *et al.* 2006; Pidoux *et al.* 2003; Shiroiwa *et al.* 2011). Mis17 may be a critical regulatory module of the Mis6 complex; however, the function of Mis17 is largely enigmatic (Shiroiwa *et al.* 2011). The carboxy-half of Mis17 is functional, because overexpression of carboxy-half of Mis17 rescued the temperature sensitivity of the *mis17-362* mutant. Contrarily, its amino-half is regulatory. Overexpression of amino-half of Mis17 in wild type caused strong negative dominance depending on several kinases.

An efficient and cost-effective suppressor mutation identification method using next-generation sequencing of genomic DNA mixture was developed (Xu *et al.* 2018). To understand the function of Mis17 and how it is regulated, here we identified extragenic suppressors for a ts mutant of a centromeric protein, *mis17-S353P*, using the genetic suppressor screen method described above. Two groups of suppressors were discovered: one group was mapped in two ribonuclease genes: *exo2* and *pan2*. The other group was mapped in EKC/KEOPS complex genes (endopeptidase-like, kinase, chromatin-associated/kinase, putative endopeptidase, and other proteins of small size). EKC/KEOPS has been implicated in telomere maintenance (Downey *et al.* 2006; Hu *et al.* 2013), transcription (Kisseleva-Romanova *et al.* 2006), genomic instability (Oberto *et al.* 2009), bipolar bud-site selection in *Saccharomyces cerevisiae* (Kato *et al.* 2011) and biosynthesis of N6-threonylcarbamoyladenosine (t^6^A), a universal tRNA modification (Daugeron *et al.* 2011; Perrochia *et al.* 2013; Srinivasan *et al.* 2011; Wan *et al.* 2016). Recently it was reported that mutations in EKC/KEOPS complex genes cause nephrotic syndrome with primary microcephaly (Braun *et al.* 2017).

## Materials and Methods

### Strains, plasmids and media

The ts mutation responsible for *mis17-362* (S353P) was re-integrated into the *S. pombe* haploid wild-type strain 972 *h*^-^ using site-directed PCR-based mutagenesis to produce a ts mutant with a wild-type background (Xu *et al.* 2018). *mis6-G135E* (containing the responsible ts mutation, G135E, of *mis6-302*), *mis12-G52E* (containing the responsible ts mutation, G52E, of *mis12-537*) and *mis18-G117D* (containing the responsible ts mutation, G117D, of *mis18-262*) were constructed in the same way (Goshima *et al.* 2003b; Hayashi *et al.* 2004; Saitoh *et al.* 1997). *exo2* and *pan2* mutants were segregated from *mis17-S353P* revertants by crossing the corresponding *mis17-S353P* revertants with the h^+^leu1 strain. *exo2* and *pan2* mutations were confirmed by Sanger sequencing. *Δgon7* was obtained from a purchased *S. pombe* haploid deletion mutant library (Bioneer Corporation). Parental *S. pombe* strains used for visualization of Cnp1-GFP, Mis6-GFP, Mis15-GFP have also been described previously (Hayashi *et al.* 2004; Saitoh *et al.* 1997; Takahashi *et al.* 2000). YPD (1% yeast extract, 2% polypeptone, 2% D-glucose) and Edinburgh Minimal Medium 2 (EMM2) were used for culturing *S. pombe* strains, and MEA medium was used for sporulation (Forsburg and Rhind 2006).

### Suppressor screening, next-generation sequencing, and suppressor identification

Suppressor screening, next-generation sequencing of suppressor genomic DNA mixtures, and suppressor mutation identification followed the procedure described by Xu *et al.* (2018). The restrictive temperature, 36°, was selected for *mis17-S353P* for suppressor screening. 1.5 × 10^8^ cells of *mis17-S353P* were screened at 37° too, but no revertants were obtained. In total, 56 revertants were obtained from ∼7 × 10^7^ cells plated for *mis17-S353P* after incubation at the restrictive temperature, 36°, for 4 days. Genomic DNA of 26 of them was extracted. Two genomic DNA mixtures were created by combining each 13 genomic DNAs in equal amounts. DNA libraries for Illumina sequencing were generated using standard protocols (Illumina) and sequenced by Illumina with paired-end (2 × 150 bp) runs using Illumina HiSequation 2000 sequencers. Finally, 13 confident extragenic suppressors were identified using the criteria described in Xu *et al.* (2018) (Table 1).

**Table 1:** Summary of the genetic screening

Gene	Original ts mutant	Mutation site	Temperature used	Cells plated	Revertants obtained	Revertants sequenced	Distinct suppressors obtained
*mis17*	*mis17-362*	S353P	36°C	7 × 10^7^	56	26	13

### Synchronous culture

To arrest cells in mitosis, nda3-KM311 (a cold-sensitive β-tubulin mutation) containing strains were used. Cells were first cultured at the permissive temperature 30° (to ∼4 × 10^6^ cells per mL). Then they were shifted to the restrictive temperature (20°) for 8 hr.

### Immunochemistry

For TCA (trichloroacetic acid) precipitation, 10 mL of *S. pombe* cell culture (containing ∼1 × 10^8^ cells) was mixed with 1/4 volume (2.5 mL) of ice-cold 100% TCA. The resulting mixture was centrifuged, and pellets were washed with 10% TCA, followed by cell disruption with glass beads in 10% TCA. After centrifugation at 8000 rpm for 10 min at 4°, washed precipitates were resuspended in SDS sample buffer containing 1 mM phenylmethyl-sulfonyl fluoride (PMSF) and boiled at 70° for 10 min. After centrifugation at 14,000 rpm for 10 min, supernatants were loaded for SDS-PAGE. Antibodies against FLAG (Sigma), Mis17 (Shiroiwa *et al.* 2011), tubulin (TAT1; a gift from Dr. Keith Gull, University of Oxford, UK) and Cdc2 (PSTAIR; a gift from Dr. Yoshitaka Nagahama, National Institute for Basic Biology, Okazaki, Japan) were employed as primary antibodies. Custom-made 3–8% gradient Tris-Acetate gels (NuPAGE, Invitrogen) were used for Mis17, while NuPAGE 12% Bis-Tris gels were run using NuPAGE MES SDS Running Buffer for Cnp1. An LAS3000 (Fuji Film) was used for signal detection.

### RNA extraction and reverse transcription-quantitative PCR (RT-qPCR)

Total RNA from *S. pombe* cells was extracted and reverse-transcribed using a PrimeScript RT reagent kit (TaKaRa) as described previously (Nakazawa *et al.* 2018). cDNA was quantified using real-time PCR (ExiCycler; Bioneer) with SYBR Premix Ex Taq II solution (TaKaRa). PCR primer sequences are available upon request.

### Structural analysis

PDB files (PDB IDs: 4WW9 and 3ENO) of EKC/KEOPS complex structures were downloaded from the Structural Bioinformatics Protein Data Bank (RCSB PDB, http://rcsb.org) (Berman *et al.* 2000) and were visualized using a molecular visualization software, PyMOL (https://pymol.org/2/). Suppressors of *mis17-S353P* were mapped onto the structures using PyMOL based on protein sequence alignment results.

### Data availability

Illumina sequence data have been deposited in the NCBI Sequence Read Archive under BioProject ID PRJNA524233 with BioSample accessions SAMN11018817 and SAMN11018818. Strains are available upon request. Table S1 lists homologs of *S. pombe* centromeric proteins in human and *S. cerevisiae*. Figure S1 shows micrographs of *exo2* and *pan2* mutants. Figure S2 validates Mis17 protein bands detected by an anti-Mis17 antibody. Figure S3 shows Mis17 and Cnp1/spCENP-A protein levels in EKC/KEOPS complex mutants. Figure S4–S6 are sequence alignment of Bud32, Kae1 and Sua5, respectively. Figure S7 describes mRNA measurement of *mis17* and *mis6* genes in wild type and mutants. Supplemental material available at Figshare: https://doi.org/10.25387/g3.7963187.

## Results

### Construction of an integrant ts strain mis17-S353P

Fission yeast Mis17 (CENP-U ortholog, Table S1) and Mis6 (CENP-I ortholog, Table S1) form the same protein complex that binds to inner centromeres and is required for loading the CENP-A/Cnp1 protein (Hayashi *et al.* 2004; Takahashi *et al.* 2000). Temperature-sensitive (ts) mutant, *mis17-362*, displayed a characteristic segregation-defective phenotype at the restrictive temperature (36°), which resulted in large and small daughter nuclei. Mis17 S353P substitution is the responsible single mutation in the mutant (Hayashi *et al.* 2004). As the original ts mutant was generated by N-methyl-N’-nitrosoguanidine (NTG) mutagenesis, genomic DNA of the mutant strain contains mostly silent mutations in addition to the ts mutation responsible for the ts phenotypes. Elimination of these additional mutations was better for isolating suppressors through whole genome sequencing to remove any potential effect of these silent mutations on suppressor identification. Hence, the mutation was chromosomally integrated in the ‘clean’ genome of the wild-type strain (972 *h*^-^) at its endogenous locus under its native promoter using site-directed mutagenesis. Thus, we obtained the integrant, *mis17-S353P*, which was temperature sensitive as the original ts mutant.

### Extragenic suppressor isolation for mis17-S353P

A spontaneous suppressor screening procedure, followed by whole-genome sequencing method was developed to isolate suppressor mutations (Xu *et al.* 2018). We followed that procedure in this study (Table 1). In total, 13 extragenic suppressors were obtained for *mis17-S353P* (Table 2). These suppressors resided in 6 genes: 4 in *exo2*, 3 in *pan2*, 1 in *gon7*, 3 in *kae1*, 1 in *bud32* and 1 in *sua5*. Exo2 and Pan2 are ribonucleases, while Gon7, Kae1, and Bud32 are in the same complex, called EKC/KEOPS, which catalyzes N6-threonylcarbamoyladenosine (t^6^A) biosynthesis together with Sua5 (Daugeron *et al.* 2011; El Yacoubi *et al.* 2009; Perrochia *et al.* 2013; Srinivasan *et al.* 2011; Wan *et al.* 2016).

**Table 2:** Extragenic suppressors of *mis17-S353P*

Mutant gene	Product	Mutation site
*exo2*	exonuclease II Exo2	G2R
*exo2*	exonuclease II Exo2	Y798Stop
*exo2*	exonuclease II Exo2	C200Stop
*exo2*	exonuclease II Exo2	A285 frame shift
*pan2*	poly(A)-specific ribonuclease complex ubiquitin C-terminal hydrolase subunit Pan2	Q69Stop
*pan2*	poly(A)-specific ribonuclease complex ubiquitin C-terminal hydrolase subunit Pan2	W665Stop
*pan2*	poly(A)-specific ribonuclease complex ubiquitin C-terminal hydrolase subunit Pan2	S730Stop
*gon7*	EKC/KEOPS complex subunit Gon7	A44P
*kae1*	EKC/KEOPS complex N(6)-L-threonylcarbamoyladenine synthase subunit	C100Y
*kae1*	EKC/KEOPS complex N(6)-L-threonylcarbamoyladenine synthase subunit	F302L
*kae1*	EKC/KEOPS complex N(6)-L-threonylcarbamoyladenine synthase subunit	D299E
*bud32*	EKC/KEOPS complex associated ATPase Bud32	G147R
*sua5*	tRNA N6-threonyl-carbamoyl-adenosine (t6A) Sua5	G223V

### Mutations in two ribonuclease genes rescue mis17-S353P

**Figure 1:**
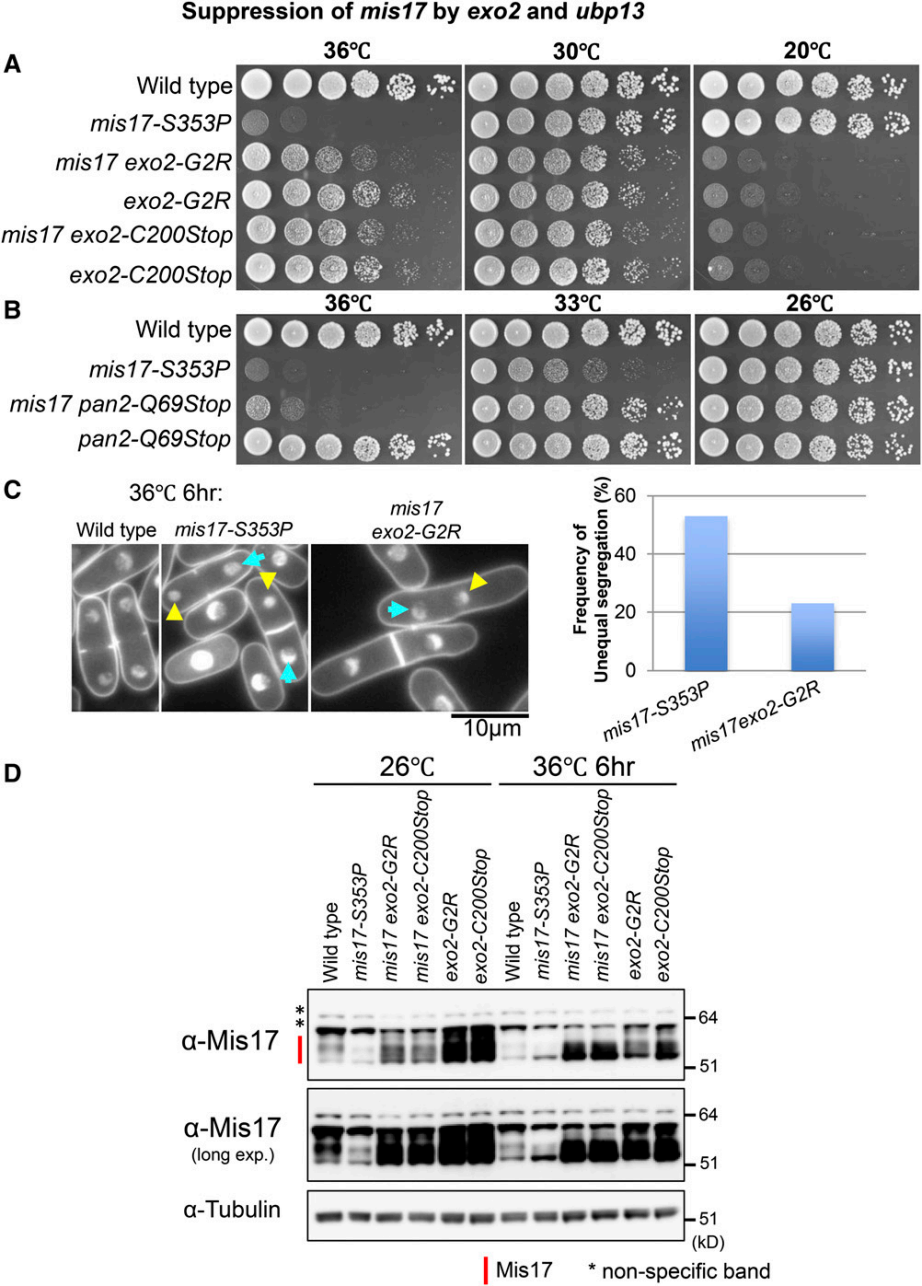
Suppression of *mis17-S353P* by *exo2* and *pan2* mutations. (A) Spot test results showing *mis17-S353P*’s suppression by *exo2* mutants (*exo2-G2R* and *exo2-C200Stop*). (B) Suppression of *mis17-S353P* by *pan2* mutant *pan2-Q69Stop*. (C) The large and small nuclei phenotype of *mis17-S353P* was suppressed by *exo2* mutants too (left panel; Large nuclei were indicated by cyan arrows and small nuclei were indicated by yellow arrowheads). Frequencies of large and small nuclei were calculated by observing 100 dividing mitotic cells for each strain (right panel). (D) Immunoblots exhibiting protein levels of Mis17 in wild type, *mis17-S353P*, *mis17 exo2* double mutants and *exo2* single mutants at both the permissive (26°C) and restrictive (36°C) temperatures. Mis17 protein bands are indicated by the red line and non-specific bands are indicated by * (Figure S2).

One group of *mis17-S353P* suppressors was located in two ribonuclease genes, *exo2* and *pan2*, products of which participate in the mRNA decay pathway (Table 2). Exo2 is a 5′-3′ exoribonuclease and its counterpart in *S. cerevisiae*, Xrn1, is required for mRNA degradation (Muhlrad *et al.* 1994). Pan2 is the ubiquitin C-terminal hydrolase subunit in the PAN complex (poly(A)-specific ribonuclease) along with Pan3 and Ppk26 (Boeck *et al.* 1996; Brown *et al.* 1996). This complex possesses poly(A)-specific ribonuclease activity to shorten mRNA poly(A) tails. Four suppressor mutations were mapped in *exo2* (G2R, C200Stop, Y798Stop and an insertion that causes an frame shift) and three mutations were mapped in *pan2* (Q69Stop, W665Stop and S730Stop, all of which are nonsense mutations). All these suppressor mutations in *exo2* or *pan2* were confirmed using the Sanger sequencing method. The *exo2-G2R*, *exo2-C200Stop* or *pan2-Q69Stop* suppressor mutations were segregated from the *mis17-S353P* revertants by crossing with wild-type strain. To confirm that suppression of the *mis17-S353P* ts phenotype was due to *exo2* or *pan2* mutations, we back-crossed *exo2-G2R*, *exo2-C200Stop* and *pan2-Q69Stop* segregants with *mis17-S353P*. The *mis17-S353P* ts phenotype was suppressed as expected (Figure 1A and 1B). *exo2-G2R* and *exo2-C200Stop* are cold sensitive (20°, Figure 1A). The N-terminus of Exo2 contains a highly conserved catalytic domain for 5′-3′ exoribonuclease. It is reported that *Δexo2* is cold sensitive too (Szankasi and Smith 1996), therefore, *exo2-G2R* and *exo2-C200Stop* may mimic the loss of Exo2 protein. Although *exo2-G2R* and *exo2-C200Stop* suppressed *mis17-S353P*, cold sensitivity of *exo2-G2R* and *exo2-C200Stop* could not be suppressed by *mis17-S353P*, therefore, the suppression is unidirectional (Figure 1A).

*mis17-S353P* exhibited unequal chromosome segregation phenotype (large and small daughter nuclei) at the restrictive temperature (Hayashi *et al.* 2004). Since *exo2* mutations strongly rescued *mis17-S353P*’s temperature sensitivity at the restrictive temperature (36°), we observed chromosome segregation phenotypes of double mutants at 36° under a fluorescence microscope after DAPI staining (Figure 1C, left panel). About 50% of mitotic dividing cells exhibited the large and small daughter nuclei phenotype in *mis17-S353P* single mutant after 6 hr of incubation at the restrictive temperature (36°) and the frequency of this large and small daughter nuclei phenotype in *mis17-S353P exo2* decreased to 23% (Figure 1C, right panel). Therefore, the unequal chromosome segregation phenotype of *mis17-S353P* was partially rescued by *exo2*.

Since *exo2-G2R* and *exo2-C200Stop* are cold sensitive, we examined phenotypes of these single segregants after 8 hr of incubation at the restrictive temperature (20°). Both *exo2-G2R* and *exo2-C200Stop* are elongated at the restrictive temperature (Figure S1A) as reported in *Δexo2* mutant cells (Szankasi and Smith 1996). *pan2* mutants are not cold sensitive and the *pan2-Q69Stop* single segregant showed no obvious phenotype (Figure S1B).

### Mis17 protein level was restored in exo2 mutants

Since Exo2 and Pan2 are involved in mRNA decay, one hypothesis to explain *mis17-S353P*’s suppression by *exo2* or *pan2* mutations is that mutant Mis17 protein is abundant in *exo2* or *pan2* mutants due to stabilization of mutant *mis17* mRNA. To test this hypothesis, we used an anti-Mis17 polyclonal antibody and verified the Mis17 protein bands by immunoblotting (Figure S2; Shiroiwa *et al.* 2011). Mis17 is a hyper-phosphoprotein showing multiple upper bands (Figure S2; Shiroiwa *et al.* 2011). As previously reported, *mis17-S353P* mutant protein level decreased dramatically at 26° and 36°, and phosphorylated bands diminished too. However, in *mis17-S353P exo2-G2R* and *mis17-S353P exo2-C200Stop* double mutants, Mis17 protein levels were significantly recovered at both temperatures (Figure 1D). Mis17 protein level is even more abundant in *exo2* single mutants. Thus, these results suggest that inhibition of the mRNA decay pathway stabilizes Mis17 protein levels.

### Suppression of mis17-S353P by mutations in EKC/KEOPS complex genes

**Figure 2:**
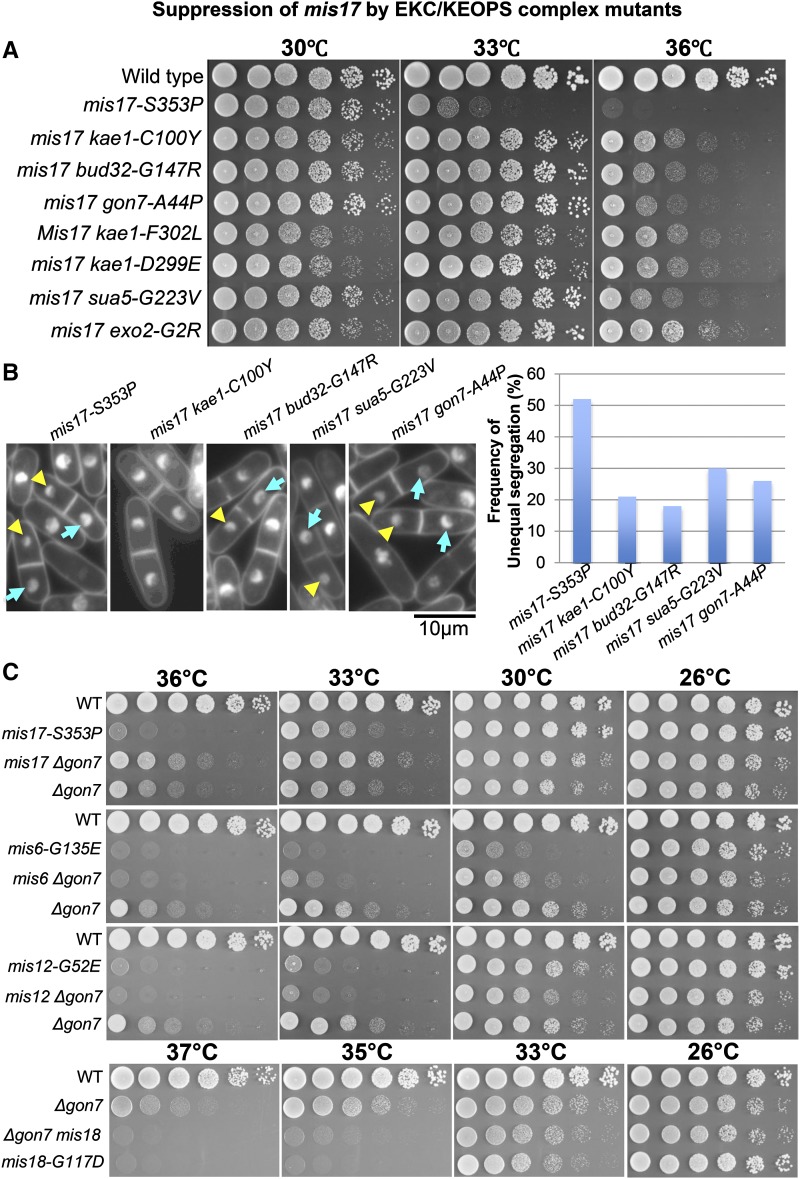
Suppression of *mis17-S353P* by EKC/KEOPS complex mutants. (A) Spot test results showing *mis17-S353P*’s suppression by EKC/KEOPS complex mutants. (B) The large and small nuclei phenotype of *mis17-S353P* at the restrictive temperature (36°C for 6hr) was suppressed by EKC/KEOPS complex mutants too (left panel; Large nuclei are indicated by cyan arrows and small nuclei are indicated by yellow arrowheads). The frequencies of large and small nuclei were calculated (right panel). (C) A *Δgon7* mutant was crossed with *mis* mutants. Double mutants rescued *mis17-S353P* and *mis6-G135E*’s temperature sensitivity too, but another two centromeric ts mutants (*mis12-G52E* and *mis18-G117D*) could not be rescued by *Δgon7*.

The other group of *mis17-S353P* suppressors was identified in EKC/KEOPS complex related genes (*gon7*, *kae1*, *bud32* and *sua5*) (Table 2 and Figure 2A). The large and small nuclei phenotype observed in *mis17-S353P* was partially rescued by these suppressor mutations (Figure 2B, left panel). The frequency of this large and small nuclei phenotype was ∼50% in *mis17-S353P* single mutant at the restrictive temperature (36°) and it decreased to 20%∼30% in double mutants (Figure 2B, right panel). Suppression of *mis17-S353P* and *mis6-G135E* by *Δgon7* suggested that loss of function of the EKC/KEOPS complex alleviated these kinetochore mutant phenotypes (Figure 2C). However, *Δgon7* could not rescue other centromeric ts mutants: *mis12-G52E* and *mis18-G117D* (Figure 2C). Mis12 and Mis18 did not belong to the Mis17-Mis6 complex (Shiroiwa *et al.* 2011), suggesting that suppression by EKC/KEOPS complex mutations might be specific to Mis17-Mis6 complex mutants. The EKC/KEOPS complex contains five subunits: Bud32, Cgi121, Gon7, Pcc1 and Kae1. It catalyzes N6-threonylcarbamoyladenosine (t^6^A) biosynthesis together with Sua5 (Daugeron *et al.* 2011; El Yacoubi *et al.* 2009; Perrochia *et al.* 2013; Srinivasan *et al.* 2011; Wan *et al.* 2016). t^6^A is a universal tRNA modification essential for normal cell growth and accurate translation (El Yacoubi *et al.* 2009; Lin *et al.* 2010; Yarian *et al.* 2002).

### Centromeric localization of Cnp1, Mis6 and Mis15 was restored

**Figure 3:**
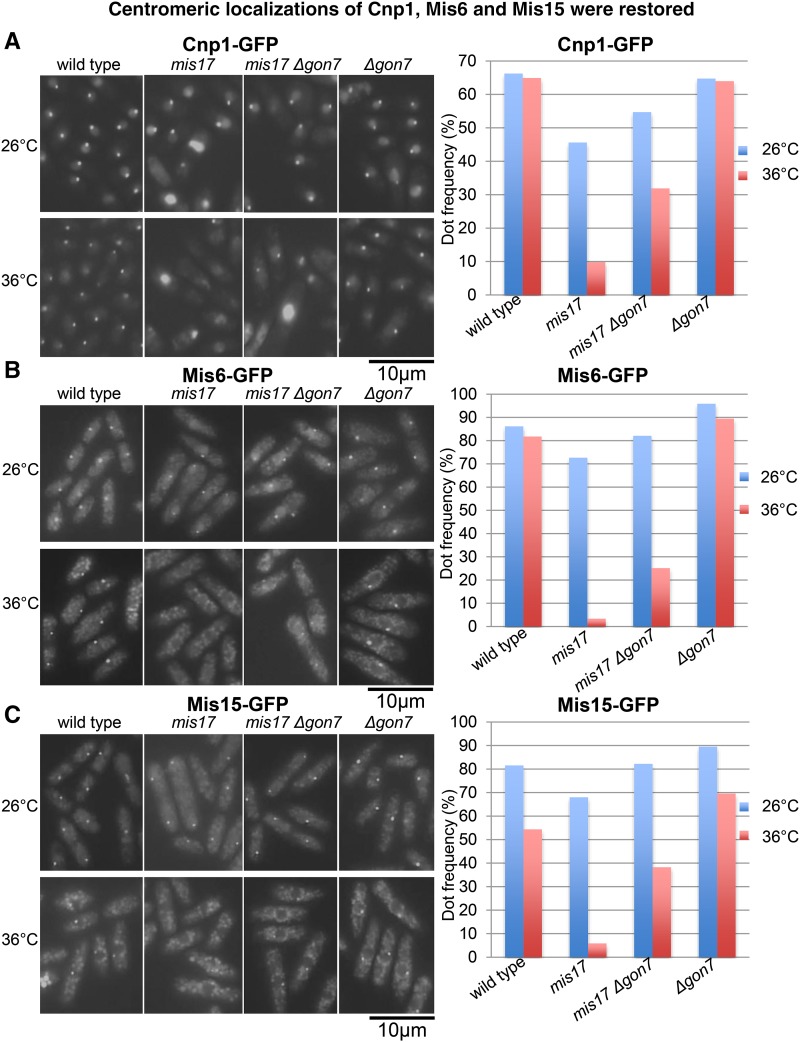
Centromeric localization of Cnp1/CENP-A, Mis6/CENP-I and Mis15/CENP-N was restored in *mis17-S353P Δgon7* double mutant. (A) Left panel, centromeric localization of Cnp1-GFP in wild type, *mis17-S353P*, *mis17-S353P Δgon7* and *Δgon7* at both the permissive (26°C) and restrictive (36°C for 5 hr) temperatures. Right panel, frequencies of kinetochore dot-like signals of Cnp1-GFP at various conditions were calculated by counting ∼200 cells in each condition. (B) Left panel, centromeric localization of Mis6-GFP at both the permissive (26°C) and restrictive (36°C for 5 hr) temperatures. Right panel, frequencies of kinetochore dot-like signals of Mis6-GFP were calculated by counting ∼200 cells in each condition. (C) Left panel, centromeric localization of Mis15-GFP at both the permissive (26°C) and restrictive (36°C for 5 hr) temperatures. Right panel, frequencies of kinetochore dot-like signals of Mis15-GFP were calculated by counting ∼200 cells in each condition.

Centromeric localization of Cnp1/CENP-A, Mis6/CENP-I and Mis15/CENP-N was impaired in *mis17-S353P* (Hayashi *et al.* 2004), therefore, we observed their localization in wild type, *mis17-S353P*, *mis17-S353P Δgon7*, and *Δgon7* at the permissive (26°) and restrictive (36°) temperatures. The kinetochore dot-like signals of Cnp1-GFP, Mis6-GFP or Mis15-GFP diminished in the *mis17-S353P* single mutant as previously reported at the restrictive temperature (left panels of Figure 3A, 3B and 3C). The loss of dot-like signals was partially rescued by *Δgon7* in *mis17-S353P Δgon7* double mutants (Cnp1-GFP, from 9 to 32%; Mis6-GFP, from 3 to 25%; Mis15-GFP, from 6 to 38%; right panels of Figure 3A, 3B and 3C). Because the Mis17-Mis6 complex is required for Cnp1/CENP-A recruitment, recovery of Cnp1/CENP-A localization at centromeres in *mis17-S353P Δgon7* double mutant indicated that the EKC/KEOPS complex may oppose the function of the Mis6-Mis17 complex in recruiting Cnp1/spCENP-A to centromeres.

### Decreased level of mutant Mis17 protein is not restored in EKC/KEOPS mutant cells

We performed immunoblots of Mis17 protein using an anti-Mis17 polyclonal antibody for wild type, *mis17-S353P* and *mis17-S353P* revertants with suppressor mutations in EKC/KEOPS complex genes at both 26° and 36°. Mis17 mutant protein level was not restored in these double mutants (Figure S3A and S3B). Therefore, the EKC/KEOPS complex does not regulate Mis17 protein level. Mutations in genes encoding the EKC/KEOPS complex rescue *mis17-S353P* by a manner distinct from that of *exo2* and *pan2* mutations.

Next, we observed Cnp1-3FLAG protein using an antibody against FLAG tag in asynchronous cells and mitotically arrested cells in wild type and two EKC/KEOPS complex mutants (*Δgon7* and *bud32-G147R*) (Figure S3C). Cnp1 protein level was not affected in these EKC/KEOPS complex mutants, suggesting that Cnp1/spCENP-A protein level or modification(s) is not regulated by the EKC/KEOPS complex.

### Loss of EKC/KEOPS complex function caused by suppressor mutations

**Figure 4:**
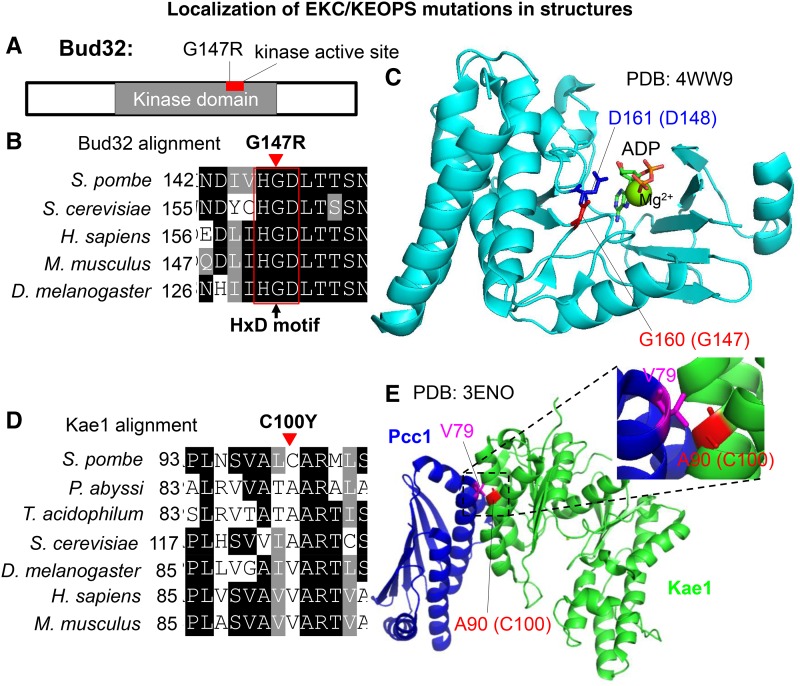
Localization of EKC/KEOPS mutations in structures. (A) Location of G147 in the kinase domain of Bud32. *bud32-G147R* was isolated as a suppressor of *mis17-S353P*. (B) G147 in HxD motif and the conservation of surrounding amino acids. (C) Localization of G147 (G160 in *S. cerevisiae*) in a Bud32 structure (PDB ID: 4WW9) from *S. cerevisiae*. (D) Conservation of amino acids surrounding Kae1 C100. *kae1-C100Y* was isolated as a suppressor of *mis17-S353P*. (E) Localization of Kae1 C100 (A90 in *T. acidophilum*) in a Kae1-Pcc1 structure (PDB ID: 3ENO).

EKC/KEOPS complex subunits are conserved among species, except for Gon7 (Figure S4–S6; Wan *et al.* 2017). Bud32 is a protein kinase (Facchin *et al.* 2002), Bud32 G147 is located in the kinase active site in the Bud32 kinase domain (Figure 4A). Bud32 G147 resides in an HRD motif, which is quite conserved and is important for proper kinase catalytic activity (Figure 4B; Mao *et al.* 2008). Bud32 D161 in *S. cerevisiae* (corresponding to Bud32 D148 in *S. pombe*) is in the catalytic cleft and directly binds ADP (Figure 4C; PDB ID: 4WW9; Zhang *et al.* 2015). Mutation of Bud32 D161 in *S. cerevisiae* destroyed its auto-phosphorylation activity at S187 and S189 (Facchin *et al.* 2002; Zhang *et al.* 2015). Bud32 G160 in *S. cerevisiae* (corresponding to Bud32 G147 in *S. pombe*) is next to D161, therefore, Bud32 G147R may cause Bud32 loss of its kinase activity in *S. pombe*. Kae1 C100 is not conserved and A or V is the corresponding amino acid in other organisms (Figure 4D). Kae1 A90 in *Thermoplasma acidophilum* (*T. acidophilum*), corresponding to *S. pombe* Kae1 C100, binds Pcc1 V79 directly (Figure 4E; PDB ID: 3ENO; Mao *et al.* 2008), therefore Kae1 C100Y mutation in *S. pombe* may disrupt the Kae-Pcc1 interaction directly (Mao *et al.* 2008; Wan *et al.* 2016).

## Discussion

Suppressor screening using ts mutant *mis17-S353P* identified two probably distinct mechanisms, affecting equal chromosome segregation. Exo2 and Pan2 may regulate chromosome segregation by restricting the Mis17 protein level, which probably is critical for centromere-specific deposition of the histone H3 variant, spCENP-A/Cnp1. Since Exo2 and Pan2 are required for mRNA degradation, we performed reverse transcription-quantitative PCR (RT-qPCR) to detect *mis17* mRNA level. *mis17* (and *mis6*) mRNA level is increased in an *exo2* mutant, although not as dramatically as expected (Figure S7).

Mutations in the EKC/KEOPS complex, which catalyzes t^6^A biosynthesis, rescued *mis17-S353P* too; however, other centromeric ts mutants (*mis12-G52E* and *mis18-G117D*), gene products of which localized at centromeres, but are not components of the Mis17-Mis6 complex, could not be rescued by an EKC/KEOPS complex mutant, *Δgon7*. Thus, our results indicate that the EKC/KEOPS complex may specifically regulates the Mis17-Mis6 complex, however further experiments are required in the future to know if other EKC/KEOPS complex mutants behave same as *Δgon7*. Considering that an excess amount of amino-half of Mis17 protein causes chromosome mis-segregation (Shiroiwa *et al.* 2011), strict control of Mis17 protein level may be crucial to prevent promiscuous deposition of spCENP-A/Cnp1 at ectopic chromosomal loci. Mis17 protein level and its phosphorylation pattern may be partly cell cycle dependent (Shiroiwa *et al.* 2011). Therefore, it is possible that the mRNA degradation pathway and the EKC/KEOPS proteins oppose the Mis17-Mis6 complex to restrict its centromeric function in a specific cell-cycle stage.

How EKC/KEOPS complex mutants rescue *mis17-S353P* (and *mis6-G135E*) is still enigmatic. Bud32 is a protein kinase (Facchin *et al.* 2002; Peggion *et al.* 2008) and *mis17-S353P* suppressor mutations in the EKC/KEOPS complex may interfere with Bud32 kinase activity or subunit-subunit interactions (Figure 4). Mis17 is hyperphosphorylated (Shiroiwa *et al.* 2011), therefore Mis17 might be a phosphorylation target of the EKC/KEOPS complex. If so, Mis17 phosphorylation may inhibit Mis17 activity at centromeres by inhibiting Mis17-Mis6 complex formation or localization.

Mis17-Mis6 complex ts mutants (*mis6*, *mis15* and *mis17*) are hypersensitive to the histone deacetylase (HDAC) inhibitor trichostatin A (TSA) (Kimata Y *et al.* 2008). N-Mis17 overproduction caused severe sensitivity to TSA too and K-to-R substitutions in the N-Mis17 fragment abolished the negative dominance effect of N-Mis17 overproduction (Shiroiwa *et al.* 2011). Whether regulation of the Mis17-Mis6 complex by the EKC/KEOPS complex is linked to the protein deacetylation-acetylation cycle in the centromeric chromatin is still unknown, and further study is required.

The Cnp1/spCENP-A protein level is not regulated by the EKC/KEOPS complex, as no change in Cnp1/spCENP-A protein level was observed when comparing wild type and an EKC/KEOPS complex mutant. Since defective localization of Mis6/CENP-I and Mis15/CENP-N at centromeres in *mis17-S353P* was partially rescued by an EKC/KEOPS complex mutant too, but the EKC/KEOPS complex mutant couldn’t rescue the temperature sensitivity of non-Mis17-Mis6 complex centromeric ts mutants (*mis12-G52E* and *mis18-G117D*), in which Cnp1/spCENP-A localization at centromeres were disrupted too. Therefore, the EKC/KEOPS complex may not directly inhibit Cnp1/spCENP-A recruitment, but presumably affects its recruitment at centromeres via Mis17-Mis6 complex formation or centromere localization.

Exo2 and Pan2 determine the stability of transcribed mRNA. The EKC/KEOPS complex has been implicated in transcription (Kisseleva-Romanova *et al.* 2006). Non-coding RNAs that are transcribed at centromeres were identified (Bayne *et al.* 2008; Choi *et al.* 2011; Djupedal *et al.* 2009; Sadeghi *et al.* 2014). Non-coding RNAs transcribed from spCENP-A/Cnp1 chromatin (central domain) are transcribed by RNA polymerase II (RNAPII) and are normally degraded by exosomes (Choi *et al.* 2011). It is also possible that mutants defective in the EKC/KEOPS complex or the mRNA decay pathway may ensure spCENP-A/Cnp1 centromeric localization by changing transcription of non-coding RNAs at centromeres, or by stabilizing RNAs.

Mutations in genes encoding protein phosphatase Ppe1 (scSit4/dmPPV/hPP6) or its bound partner Ekc1 (scSAP) were identified as suppressors of *mis12-537* (Goshima *et al.* 2003a). Suppression of *mis12* mutants by deletion of Ppe1 is quite specific, as *mis6-302* was not suppressed by *ppe1* mutation. Extragenic suppressor screening of *mis18-818* and *mis19-1* isolated two groups of loss-of-function suppressor mutations in non-sense-mediated mRNA decay factors (*upf2* and *ebs1*), and in SWI/SNF chromatin-remodeling components (*snf5*, *snf22* and *sol1*) (Hayashi *et al.* 2014). With the suppressor screening done in this study, we find no overlap among these suppressor genes. Therefore, although all Mis proteins are centromeric proteins and their ts mutants all exhibited large and small nuclei phenotype at the restrictive temperature, the Mis17-Mis6 complex, the Mis16-Mis18-Mis19-Mis20 complex, and Mis12 are regulated distinctively. Further study is required to address molecular mechanisms restricting the Mis17-Mis6 complex’s centromeric function through the mRNA decay pathway and the EKC/KEOPS complex.
